# Symptoms of Autism Spectrum Disorder in Individuals with Down Syndrome

**DOI:** 10.3390/brainsci11101278

**Published:** 2021-09-26

**Authors:** Amanda Dimachkie Nunnally, Vivian Nguyen, Claudine Anglo, Audra Sterling, Jamie Edgin, Stephanie Sherman, Elizabeth Berry-Kravis, Laura del Hoyo Soriano, Leonard Abbeduto, Angela John Thurman

**Affiliations:** 1 MIND Institute, University of California Davis Health, 2825 50th Street, Sacramento, CA 95816, USA; vivng@ucdavis.edu (V.N.); Claudine.Anglo@ucsf.edu (C.A.); ldelhoyo@ucdavis.edu (L.d.H.S.); ljabbeduto@ucdavis.edu (L.A.); ajthurman@ucdavis.edu (A.J.T.); 2 Waisman Center and Department of Communication Sciences and Disorders, University of Wisconsin-Madison, Madison, WI 53706, USA; audra.sterling@wisc.edu; 3 Department of Psychology, University of Arizona, Tucson, AZ 85721, USA; jedgin@email.arizona.edu; 4 Department of Human Genetics, School of Medicine, Emory University, Atlanta, GA 30322, USA; ssherma@emory.edu; 5 Departments of Pediatrics, Neurological Sciences and Biochemistry, Rush University Medical Center, Chicago, IL 60612, USA; elizabeth_berry-kravis@rush.edu; 6 Department of Psychiatry and Behavioral Sciences, University of California, Davis Health, Sacramento, CA 95817, USA

**Keywords:** Down syndrome, autism spectrum disorder, co-occurring, prevalence

## Abstract

There is a growing body of evidence to suggest that individuals with Down syndrome (DS) are diagnosed with autism spectrum disorders (ASD) at a higher rate than individuals in the general population. Nonetheless, little is known regarding the unique presentation of ASD symptoms in DS. The current study aims to explore the prevalence and profiles of ASD symptoms in a sample of individuals with DS (*n* = 83), aged between 6 and 23 years. Analysis of this sample (*M_Age_* = 15.13) revealed that approximately 37% of the sample met the classification cut-off for ASD using the Autism Diagnostic Observation Schedule 2 (ADOS-2) Calibrated Severity Score (CSS), an indicator of the participants’ severity of ASD-related symptoms. Item-level analyses revealed that multiple items on Module 2 and Module 3 of the ADOS-2, mostly in the Social Affect (SA) subdomain, differentiated the children with DS who did not meet ASD classification (DS-only) from those who did (DS + ASD). Lastly, comparisons of individuals with DS-only and those with DS + ASD differed significantly on the syntactic complexity of their expressive language. These findings shed light on the unique presentation of ASD symptoms in a sample of individuals with DS and suggest that expressive language abilities may play a pivotal role in the presentation of ASD symptoms in DS.

## 1. Introduction

Down syndrome (DS) is caused by the presence of a third copy of all or part of chromosome 21 and is the leading genetic cause of intellectual disability (ID), affecting approximately 1 in 700 individuals born in the United States [1]. Individuals with DS have historically been described as particularly affable and sociable [2], leading to the belief that they do not experience substantial challenges in the social domain. This belief, however, has been challenged by findings of delays in the development of social communication and social cognition associated with DS, detected as early as infancy [3,4,5,6,7,8,9]. In addition to social communication delays in this population, researchers have also reported higher rates of restricted and repetitive interests and behaviors [5,6]. Although the combination of challenges in social communication and rigid and repetitive interests and behaviors is most often associated with autism spectrum disorders (ASD), there is also research to suggest that these symptoms also present among individuals with DS at low risk for ASD [5,6], likely as a reflection of the cognitive and linguistic delays associated with the DS phenotype [10,11,12]. Studies are needed to clarify whether the social affective challenges and restricted and repetitive interests and behaviors in individuals with DS are best viewed as symptoms of ASD or of the DS phenotype more generally.

Although variable findings regarding the prevalence of ASD among individuals with DS have been reported (16–42%) [13,14,15], nearly all studies have reported a prevalence higher than the 1.9% (i.e., 1 in 54) prevalence rate observed in the general population [16]. The studies reporting on the prevalence of ASD symptomatology among individuals with DS have differed in the instruments used to ascertain symptoms in this population, with studies utilizing direct assessment methods (e.g., Autism Diagnostic Observation Schedule-2 (ADOS-2) [17]) and/or parent report measures (e.g., Autism Diagnostic Interview-Revised (ADI-R) [17,18,19], Social Communication Questionnaire (SCQ) [3,15,20], Social Responsiveness Scale (SRS) [5,6], and Aberrant Behavior Checklist (ABC) [21,22]). These data provide a starting point for understanding the nature of social affective skills and restricted and repetitive interests and behaviors among individuals with DS.

There is a relatively small body of work that has demonstrated differences in the presentation of social communication challenges and rigid and repetitive interests and behaviors when comparing individuals with DS + ASD with individuals with DS without co-occurring ASD (referred to hereafter as DS-only). More specifically, researchers using the Aberrant Behavior Checklist (ABC) have found that individuals with DS + ASD present with higher levels of stereotypy and repetitive behaviors than their counterparts with DS-only [21,22]. Similar results were presented by researchers using the ADI-R to compare ASD symptomatology in individuals with DS and those with DS + ASD, finding that individuals with DS + ASD had elevated scores on the reciprocal social interaction, communication and restricted, repetitive, and stereotyped patterns of behavior subdomains when compared to their peers with DS matched on mental-age (MA) [18,19]. These findings have also been replicated using direct observation measures, including in the only study to use the ADOS-2, finding more rigid and repetitive behaviors and greater social communication challenges among individuals with DS + ASD in comparison to those with DS-only [17]. Collectively, these findings suggest more severe ASD symptomatology among individuals with DS + ASD in comparison to those with DS-only. Additionally, although differences have been found between the ASD symptomatology exhibited by individuals with DS + ASD and those with DS-only, it is important to note that studies have found that individuals with DS-only still present with elevated rates of ASD symptomatology when comparing their scores to normative sample means [5,6].

In addition to differences in ASD symptomatology among individuals with DS-only and DS + ASD, group differences have also been found in terms of other dimension of functioning. First, individuals with DS + ASD have been found to have lower cognitive abilities when compared to those with DS-only [5,17,19]. Additionally, differences in expressive and receptive language abilities have been reported, such that individuals with DS + ASD have lower expressive and receptive language abilities than those with DS-only [17,19]. These findings further bolster the notion that individual differences in important domains of ability, such as language, may play a role in the presentation of ASD symptomatology among individuals with DS + ASD.

Altogether, this body of research begins to provide an understanding of the overall nature of social affective skills and restricted and repetitive behaviors and interests among individuals with DS; however, further research is needed to better understand the specific profile of ASD symptomatology in this population. Improving understanding of the presentation of ASD in individuals with DS could lead to earlier and more accurate ASD classification in this population, allowing for earlier access to intervention services which could improve long-term outcomes. Furthermore, the ability to discriminate between symptoms and behaviors that are phenotypic of DS and those which are indicative of ASD would be helpful in determining the type of intervention needed to facilitate improved outcomes.

The purpose of the current study was to use a direct assessment, gold-standard autism diagnostic instrument (i.e., the ADOS-2) in a sample of individuals with DS to: (1) explore the proportion of individuals who meet criteria for ASD diagnosis, (2) determine whether individuals who do and do not meet criteria for ASD diagnosis differ on key individual characteristics such as cognitive and linguistic ability, and (3) investigate whether specific items on Module 2 and Module 3 of the ADOS-2, which are designed to be administered to individuals at different developmental levels, differentiated those who met criteria for ASD diagnosis from those who did not.

## 2. Materials and Methods

Study procedures were reviewed and approved by Institutional Review Boards at all participating universities. Written informed consent was obtained from participants’ guardians, and verbal assent was obtained from the youth prior to beginning study procedures. All data for the present study were collected at participants’ initial visit.

### 2.1. Participants

The sample for the current study was drawn from a larger sample of individuals with DS, aged between 6 and 23 years, who were recruited as part of a multi-site study evaluating the feasibility of expressive language sampling (ELS) as an outcome measure [23,24]. The chronological age range of the larger ELS project was selected to include individuals who would likely be able to meaningfully complete the ELS tasks and exclude those who might display clinically significant signs of Alzheimer’s Disease. All participants provided medical documentation of Down syndrome (i.e., trisomy 21 or translocation) without mosaicism, and all met criteria for ID. In addition, the following inclusion criteria were utilized in the larger study, based on parent report: (1) participant and caregiver willingness to partake in the protocol; (2) participants’ use of speech as their primary mode of communication, with the use of at least occasional multi-word utterances; (3) participants’ use of English as their primary language; (4) no more than mild hearing loss; (5) no serious (uncorrected) visual impairment that may interfere with participants’ performance on the testing battery; (6) participants’ IQs fell within the range for ID (≤70) and (7) participants were not enrolled in a randomized control trial or experiencing medication, treatment or significant educational changes during the 8 weeks prior to the initial testing visit.

In the larger study, participants were recruited and tested at four university sites, located in Arizona, Georgia, California and Wisconsin, although many participants resided outside these states. The total sample of 107 participants with DS (55 males, 52 females; *M_Age_* = 15.13). Participants in the present study were excluded from or analysis if they had missing or incomplete ADOS-2 (*n* = 13) or if they received Module 1 of the ADOS-2 (*n* = 6). The decision to exclude participants who received Module 1 of the ADOS-2 from analyses was based on the study inclusion criteria, which was meant to target participants with at least multi-word utterances. Because these eligibility criteria would have excluded most participants with DS for whom Module 1 of the ADOS-2 was chosen, as they would not meet the threshold for language, these data were not considered representative of the larger population. The final sample for the present study was thus comprised of 83 participants (45 males, 38 females) with a mean age of 15.54 years (*SD* = 5.19) and a mean Stanford-Binet Intelligence Scales, Fifth Edition (SB-5) Full Scale IQ deviation score of 46.65 (*SD* = 11.21). The choice to use deviation scores in this study was due to the large number of participants that received the lowest possible score (floor scores) on FSIQ. Deviation scores provide z-score transformations based on population norms and are useful in ameliorating floor effects and allow for a more accurate measure of cognitive abilities among individuals with ID [25].

Because some of the research objectives consider participant performance as a function of ADOS-2 module, participant characteristics were also considered as a function of module. Participants who received Module 2 were, on average, younger than those who received Module 3 (*F*(1,81) = 16.339, *p* < 0.001). Additionally, lower VIQ deviation scores (*F*(1,73) = 37.748, *p* < 0.001) and NVIQ deviation scores (*F*(1,76) = 22.663, *p* < 0.001) were observed for participants receiving Module 2 in comparison to those receiving Module 3. Conversely, participants who received Module 2 and those who received Module 3 were not significantly different in their adaptive functioning skills (*F*(1,64) = 656, *p* = 0.421). Please see Table 1 for additional details.

### 2.2. Measures

#### 2.2.1. Cognitive Ability

Participants’ cognitive ability was assessed using the Stanford Binet Intelligence Scales, Fifth Edition (SB-5) [26]. Deviation scores were calculated to provide descriptive information on the study sample for Full Scale IQ (FSIQ), Non-Verbal IQ (NVIQ), Verbal IQ (VIQ), following procedures outlined by Sansone and colleagues [26]. Deviation scores, which provide z-score transformation based on the general population norms, are helpful in mitigating floor effects and lend themselves to a more precise measurement of cognitive ability in populations with ID [25]. In addition, Non-Verbal Change Sensitive scores, the equivalent of growth scores, were calculated for use in study analyses.

#### 2.2.2. ASD Symptom Severity

The Autism Diagnostic Observation Schedule-2 is a semi-structured, standardized play-based assessment used to measure reciprocal interactions and repetitive behaviors. Participants in this sample received either Module 2 (*n* = 45) or Module 3 (*n* = 38) of the ADOS-2, administered by examiners trained to research reliability. In addition, site examiners scored video administrations and participated in cross-site pre-collection reliability calls to calibrate scoring and cross-site reliability was also assessed for 13 DS administrations collected on the project. Administrator reliability was 86% for all items and 87% for algorithm items. ADOS-2 modules were assigned based on participants’ verbal ability following the published guidelines for the measure, such that participants with “phrase speech up to fluent speech” received Module 2, and those who are “producing a range of flexible sentence types, providing language beyond the immediate context, and describing logical connections within a sentence” [27] (p. 10) received Module 3. Due to the level of developmental delay exhibited by participants, no participants demonstrated “a minor level of independence in relationships and goals” [27] (p. 11) required to receive Module 4 of the ADOS-2. For the purposes of this study, the overall calibrated severity score (Overall CSS), Social Affect calibrated severity score, (SA-CSS), and Restricted and Repetitive Behavior calibrated severity score (RRB-CSS) were calculated to provide standardized scores for symptom severity [28]. Both the Overall CSS and SA-CSS are assessed using a 10-point scale. In contrast, the RRB-CSS score is assessed using a 7-point scale that was spread across a 10-point scale range, in which the scores 2, 3, and 4 are not possible to obtain [29]. Participants’ ASD classification was determined using Overall CSS, in accordance with procedures outlined by Gotham, Pickles and Lord [28]. Finally, for participants who were older than the norming sample of the ADOS-2, the upper age limit of the CSS norming tables was used to compute CSSs.

#### 2.2.3. Expressive Language Sampling

Participants’ expressive syntactic and lexical levels were assessed using a narration task in which participants were asked to narrate a story using a wordless picture book [23,24]. The task begins with the participant familiarizing themselves with the book by examining each page spread for approximately 10 s before narrating the story depicted in the book. The examiner facilitates the narration by controlling the book and waiting for the participant to finish their description before turning the page. In order to standardize the task, participants received one of two books from the Mercer Mayer’s “Frog” series (“Frog Goes to Dinner” or “Frog on His Own”), and administrators relied on a standardized set of prompts and responses to ensure minimal and consistent scaffolding across participants. Participants’ speech was transcribed, segmented into C-units (communication units), with a C-unit is defined as an independent clause with associated modifiers, including dependent clauses, and analyzed using the software program Systematic Analysis of Language Transcripts 18 Research (SALT) [30]. Inter-transcriber agreement data were computed and averaged as follows: 87% for utterance segmentation, 87% for identification of partly or fully unintelligible C-units, 84% for identification of the exact lexical and morphemic content of each C-unit, 76% for identification of the exact number of morphemes in each C-unit and 80% for the exact number of words in each C-unit [24]. Construct validity has been established for the ELS narration task such that medium to strong convergent validity was found with directly administered and informant report measures for similar constructs measuring syntactic complexity and lexical diversity in individuals with DS as well as other forms of ID [23,24].

*Syntactic complexity.* Participants’ syntactic maturity was assessed by calculating the mean number of morphemes per C-unit. Only complete and fully intelligible C-units were used to calculate this variable.

*Lexical Diversity.* The size of each participant’s expressive vocabulary was computed by calculating the number of different word roots in the participant’s first 50 complete and fully intelligible C-units. In the event that the participant produced less than 50 complete and fully intelligible C-units, the full sample was used.

### 2.3. Data Analysis

To address Objective 1, the frequency of ASD classification (i.e., the number of individuals who had an overall CSS that met the cutoff for ASD) is presented for the overall sample, as well by module. One-way ANOVAs were conducted to compare the frequency of classification in Module 2 and Module 3. Additionally, distributions for CSS on the SA and RRB subdomains were also presented for the overall sample, and one-way ANOVAS were conducted to determine whether group differences (DS + ASD vs. DS-only) between SA-CSS and RRB-CSS were detected in the overall sample. Next, to address Objective 2, analyses compared key individual characteristics (chronological age, nonverbal change sensitive score, syntactic complexity and lexical diversity) between participants with DS who did not receive an ASD classification on the ADOS-2 (DS-only) and participants with DS who received an ASD classification on the ADOS-2 (DS + ASD). Parametric analyses (one-way ANOVAs) were used to address Objective 3 due to the continuous nature and normal distribution of participant characteristic. Lastly, to address Objective 3, we explored group differences between individuals with DS-only and DS + ASD across ADOS-2 algorithm items, doing so separately for each of the ADOS-2 modules since items differ between Module 2 and Module 3 of the ADOS-2. Because of the ordinal nature and non-normal distribution of ADOS-2 algorithm item scores, nonparametric analyses (Mann–Whitney U-Tests) were used. False Discovery Rate (FDR) corrections were applied within each set of analyses, in accordance with procedures outlined by Benjamini and Hochberg [31], to maintain a family-wise alpha rate of *p* ≤ 0.050.

## 3. Results

### 3.1. Prevalence of ASD

#### ASD Classification

**Figure 1:**
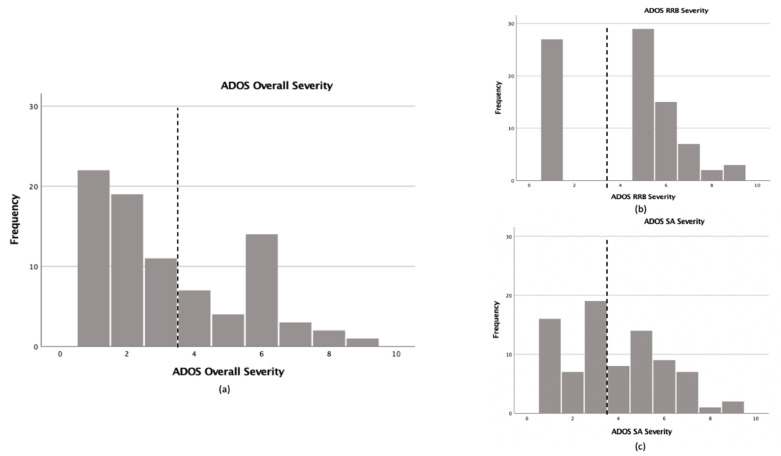
Frequency distribution of ADOS-2 CSSs across sample; (**a**) ADOS-2 Overall CSS for overall sample; (**b**) ADOS-2 RRB-CSS for overall sample; (**c**) ADOS-2 SA-CSS for overall sample. Note: Dotted line represents CSS cutoff for ASD classification.

In the current sample, 37.3% of participants met the overall classification criteria for ASD on the ADOS-2 (i.e., DS + ASD). The prevalence of ASD was higher among those receiving Module 2 (46.7%) than among those receiving Module 3 (26.3%); statistical comparisons indicated that this difference in rate between modules approached significance (*F*(1,82) = 3.722; *p* = 0.057; η^2^ = 0.044). See Figure 1 for distribution of participant scores.

**Figure 2:**
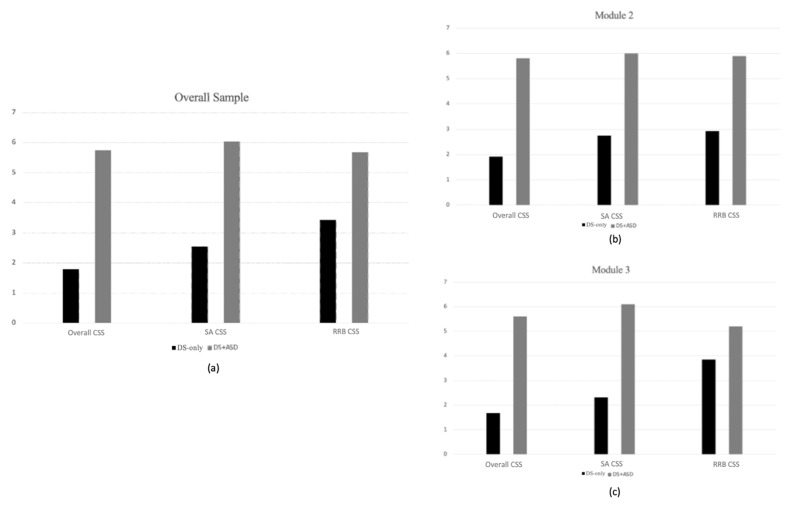
Group differences in mean ADOS-2 CSSs; (**a**) Group differences in mean ADOS-2 CSSs for overall sample; (**b**) Group differences in mean ADOS-2 CSSs for Module 2; (**c**) Group differences in mean ADOS-2 CSSs for Module 3.

Analyses were also conducted to determine whether participants classified as DS + ASD differed from participants classified as DS-only on CSS scores for the SA and RRB subdomains. In the overall sample, group differences were detected in both the SA (*F*(1,82) = 151.740; *p* < 0.001; η^2^ = 0.652) and RRB domains (*F*(1,82) = 20.115; *p* < 0.001; η^2^ = 0.199), such that individuals with DS + ASD had higher scores than those with DS-only. When exploring group differences at the module level, significant differences were detected for both SA-CSS (*F*(1,44) = 76.495; *p* < 0.001; η^2^ = 0.640) and RRB-CSS for Module 2 (*F*(1,44) = 35.350; *p* < 0.001; η^2^ = 0.451); however, only SA-CSS significantly differentiated groups in Module 3 (*F*(1,37) = 64.184; *p* < 0.001; η^2^ = 0.641). Please see Figure 2 for means.

### 3.2. Group Differences across Characteristics

We compared the participants classified as DS + ASD to participants classified as DS-only in terms of chronological age, nonverbal cognitive ability, lexical diversity, and syntactic complexity (see Table 2). Significant group differences were found for nonverbal cognitive ability (*F*(1,82) = 1.091; *p* = 0.044; η^2^ = 0.053), lexical diversity (*F*(1,82) = 7.330 *p* = 0.008; η^2^ = 0.085), and syntactic complexity (*F*(1,82) = 4.198; *p* = 0.000; η^2^ = 0.168), such that group means were lower for participants classified as DS + ASD than for participants classified as DS-only across all comparisons. The differences in lexical diversity and syntactic complexity, but not nonverbal cognitive ability, remained significant after applying the FDR correction.

Follow up analyses were conducted to determine whether differences found in lexical diversity and syntactic complexity were also seen for each of the modules analyzed separately. Results of these analyses revealed that, within Module 2, participants classified as DS + ASD produced C-units with less syntactic complexity (*F*(1,42) = 7.095 *p* = 0.011; η^2^ = 0.148) than participants classified as DS-only. Similar results were found for Module 3, with a lower mean for syntactic complexity (*F*(1,37) = 5.201 *p* = 0.029; η^2^ = 0.126) found for participants classified as DS + ASD than DS-only. No significant differences in lexical diversity was found between individuals who were classified as DS + ASD and those classified as DS-only in either Module 2 (*F*(1,42) = 2.484 *p* = 0.123; η^2^ = 0.057) or Module 3 (*F*(1,37) = 1.125 *p* = 0.296; η^2^ = 0.030).

### 3.3. Group Differences across ADOS-2 Items

#### 3.3.1. Module 2 Items

**Figure 3:**
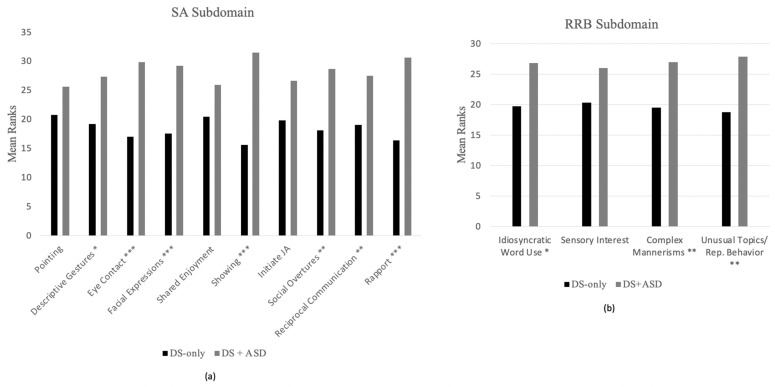
Mean Rank comparisons for Module 2 algorithm items; (**a**) Mean Rank comparisons for Module 2 SA items; (**b**) Mean Rank comparisons for Module 2 RRB items. Note: JA = Joint attention. * *p* < 0.05; ** *p* < 0.01; *** *p* < 0.001.

Between-group comparisons of algorithm items on Module 2 of the ADOS-2 revealed 7 items in the SA domain and 3 items in the RRB domain that differentiated participants with DS + ASD from participants with DS-only (see Figure 3). The differences on all of these items remained significant after the FDR correction. More specifically, differences in group mean ranks were detected for SA algorithm items measuring: (1) descriptive, conventional, instrumental or informational gestures (U = 160.50; *p* = 0.012); (2) unusual eye contact (U = 108.00; *p* < 0.001); (3) facial expressions directed to others (U = 122.00; *p* = 0.001); (4) showing (U = 74.50; *p* < 0.001); (5) quality of social overtures (U = 133.50; *p* = 0.002) ; (6) amount of reciprocal social communication (U = 157.50; *p* = 0.017); (7) overall quality of rapport (U = 92.50; *p* < 0.001). Additionally, differences in group mean ranks were detected for RRB algorithm items measuring: (1) stereotyped/Idiosyncratic use of words or phrases (U = 172.50; *p* = 0.029); (2) hand and finger and other complex mannerisms (U = 188.50; *p* = 0.002); (3) unusually repetitive interests or stereotyped behaviors (U = 150.50; *p* = 0.007).

#### 3.3.2. Module 3 Items

**Figure 4:**
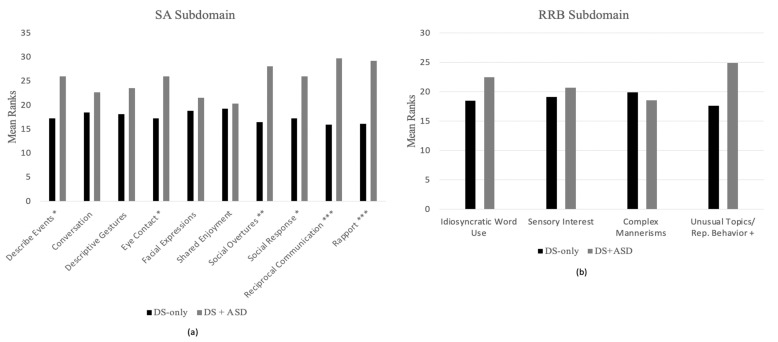
Mean Rank comparisons for Module 3 algorithm items; (**a**) Mean Rank comparisons for Module 3 SA items; (**b**) Mean Rank comparisons for Module 3 RRB items. Note: * *p* < 0.05; ** *p* < 0.01; *** *p* < 0.001; + signifies no longer significant after FDR correction.

Between-group comparisons were also conducted for algorithm items on Module 3 of the ADOS-2 (see Figure 4). Group differences were found in the mean ranks for the five SA algorithm items measuring: (1) reporting of events (U = 75.500; *p* = 0.016); (2) unusual eye contact (U = 76.00; *p* = 0.005); (3) quality of social overtures (U = 54.50; *p* = 0.001); (4) quality of social response (U = 76.00; *p* = 0.017); (5) amount of reciprocal social communication (U = 38.00; *p* < 0.001); (6) overall quality of rapport (U = 43.00; *p* < 0.001). These differences remained significant even after applying the FDR corrections. One algorithm item from the RRB subdomain, measuring unusually repetitive interests or stereotyped behaviors, emerged as significantly different between participants who met classification from those who did not (U = 86.50; *p* = 0.034); however, this finding did not remain significant after applying the FDR correction.

## 4. Discussion

The detection of co-occurring ASD among individuals with ID associated with known genetic conditions, such as DS, poses many challenges [32,33]. It is becoming increasingly apparent that individuals with DS are at increased risk for presenting with the symptoms ASD relative to the general population [5,6]; moreover, due to the developmental delays associated with the DS phenotype may influence the presentation of ASD symptomatology in this population [10,11,12]. Studies that clarify the nature of social communication skills and restricted and repetitive interests and behaviors in DS, and other populations with ID, can provide important information for understanding the unique way in which ASD presents among individuals. The goal of the present study was to elucidate the prevalence and the factors shaping the presentation of ASD symptomatology in a large sample of individuals with DS using the ADOS-2, a gold standard direct-assessment diagnostic instrument.

### 4.1. ASD Classification

Several key findings emerged from the present study. First, we explored the prevalence of ASD in a sample of 83 individuals with DS and found that 37.3% of the sample met overall classification criteria for ASD on the ADOS-2, which falls within the range of prevalence rates presented in several previous publications on DS [13,14,15]. This similarity in prevalence rates suggests that the specific measures used may not differ significantly in their utility in detecting ASD in individuals with DS. Individuals in the sample who had overall scores that met classification cutoff for ASD (DS + ASD) had significantly higher SA-CSS and RRB-CSS scores than individuals who did not (DS-only). This finding is consistent with prior research finding of both more challenges in social communication and increased rigidity and repetitive behaviors when comparing individuals classified as having DS-only and those classified as having DS + ASD [18,19,20,21,22]. It should be noted that studies have found that individuals with DS who were at low risk for ASD nonetheless presented with challenges in social communication and restricted and repetitive interests and behaviors relative to normative expectations for their chronological ages, indicating that these symptoms and behaviors may also be phenotypic to DS [5,6]. This is further underscored by findings in the current study that a number of individuals received overall scores on the ADOS-2 that were right below the cutoff for ASD classification.

Closer examination of the data at the module level indicated a trend for overall ASD classification rates to be almost two times higher among those individuals receiving the ADOS-2 Module 2 than on those receiving Module 3. Group differences were detected in Module 2, with individuals with DS + ASD having significantly higher means on both the SA-CSS and RRB-CSS than those with DS-only. There are two possible explanations for the differences seen in ASD classification rates and extent of the differences between individuals receiving the two modules. First, the finding of overall lower cognitive and linguistic abilities amongst individuals who received Module 2, which has been shown to be related to ASD symptomatology in this population could be driving this effect [5,17,18]. Second, it is possible that particular items, activities, and/or norming procedures associated with the Module 2 are contributing to these differences [34,35,36]. In other words, the items specific to Module 2 may be more sensitive to the comparisons made in the present study, therefore, considering item analyses and the influence of participant characteristics on classification rates provide a start to clarifying these findings. In addition to differences in classification rates between modules, differences emerged such that for individuals who received Module 3, only the SA-CSS differentiated individuals with DS + ASD from those with DS-only, with the former having significantly higher scores. There may be several explanations for the RRB-CSS not differentiating groups in Module 3. First, since fewer individuals receiving Module 3 had an RRB-CSS that met the cutoff for ASD classification, between-group comparisons within Module 3 could be underpowered. Second, stereotyped behaviors, highly restricted interests, rigidity, and inflexibility are common in DS [5,18] and may be best viewed as an inherent part of the DS phenotype rather than being reflective of ASD. Given the overlap between seemingly phenotypic rigid and repetitive behaviors seen among individuals with DS and those associated with the core deficits of ASD, further research is necessary to better understand whether these behaviors can be considered indicative of co-occurring ASD in this population or whether they instead reflect different underlying mechanisms and challenges.

### 4.2. Group Differences across Individual Characteristics

We explored whether differences in chronological age, nonverbal cognitive abilities, and language skills differentiated individuals who were classified as DS + ASD from those classified as DS-only. It is important to note that the group classification was based on ADOS-2 cutoff scores and does not imply a formal (clinical) diagnosis of ASD. Instead, these groups simply reflect individuals’ presentation of ASD symptomatology on the ADOS-2. With that said, when examining groups differences in the overall sample, significant differences (after correcting for multiple comparisons) were identified in lexical diversity and syntactic complexity were detected, such that individuals with DS-only performed at a higher level than those with DS + ASD. Results of follow up analyses indicated that while syntactic complexity significantly differentiated individuals with DS + ASD from those with DS-only in both Module 2 and Module 3, lexical diversity was not a significant difference at the individual module level. One possible explanation for the finding that lexical diversity differentiated groups in the overall sample but not at the module level may be that the difference detected may be more appropriately attributed to ID associated with DS than to ASD symptomatology. This is supported by the fact that individuals who received Module 2 had, on average, lower cognitive and linguistic abilities than those who received Module 3. Overall, the finding of lower linguistic abilities among individuals with DS + ASD in the overall sample is consistent with previously reported findings of lower verbal abilities among individuals with DS + ASD than those with DS-only [19,21]. Moreover, these findings underscore the need to consider the linguistic delays characteristic of individuals with DS in the interpretation of ADOS-2 algorithm items, especially with samples of older individuals with ASD, as studies to derive algorithm items were originally normed using samples of individuals aged up to 12 years [34,35].

### 4.3. Group Differences across ADOS-2 Items

We explored the presentation of ASD symptomatology in this sample by comparing groups (i.e., DS + ASD and DS-only) across algorithm items of the ADOS-2. Analyses were conducted at the module level because algorithm items differ between modules. Items on the SA subdomain of the ADOS-2 were more likely to differentiate individuals with DS + ASD from those with DS-only, with four common items emerging across modules: unusual eye contact, quality of social overtures, amount of reciprocal social communication and overall quality of rapport. Of note, all four significant algorithm items in the SA subdomain rely on pragmatic social communication skills, which have been found to be delayed among young children with DS and other forms of ID relative to normative age expectations. In previous studies, children with DS have also been found to have a relative weakness in pragmatics in comparison with their structural language abilities [37]. Additionally, the finding that unusual eye contact differentiated the groups is of interest given that atypical eye contact in DS has been detected as early as infancy [4] and the use of eye contact among children with DS is less clear than among their typically developing (TD) peers and peer with other developmental disabilities (DD) [38]. These results suggest that challenges in pragmatic social communication are characteristic of the DS phenotype and indicate the need to clarify boundaries between standard phenotype heterogeneity from comorbid ASD symptomatology.

At the same time, many commonalities were observed among SA items that did not differentiate the two DS groups. In Module 2, items measuring pointing, shared enjoyment and joint attention did not significantly differentiate individuals classified as DS-only from those classified as DS + ASD. Similarly, the following four SA items from Module 3 did not significantly differentiate the groups: conversation, descriptive gestures, facial expressions and shared enjoyment. Notably, shared enjoyment did not discriminate between groups in either module, a finding that is not entirely surprising given that, although individuals with DS may struggle with certain aspect of social communication, findings suggest relative strengths in social engagement and social orientation (i.e., sociability) [39]. Additionally, in Module 2, joint attention abilities did not differentiate between participants’ group membership. This finding is consistent with literature finding that joint attention may be a relative strength among individuals with DS, who perform similarly to children with TD and at a higher level than children with ASD and other NDDs [40]. Although joint attention (JA) was not explicitly measured in Module 3, it could be argued that joint attention is a developmental antecedent to the use of descriptive gestures, which is measured in Module 3 [41]. The finding that the item measuring the use descriptive gesture also did not differentiate groups may suggest that the use of JA may continue to be a strength among individuals with DS throughout childhood. These results suggest that some phenotypic characteristics of DS, such as relative strengths in social orientation and joint attention, may offset the presentation of these skills among individuals with DS + ASD. Further research is needed to better understand how phenotypic characteristics of DS affect ASD symptom presentation among individuals with DS + ASD.

Three items from the RRB subdomain of the ADOS-2 (stereotyped/idiosyncratic use of words and phrases, hand, finger and other complex mannerisms and unusually repetitive interests or stereotyped behaviors) significantly differentiated individuals with DS-only from those with DS + ASD in Module 2, and (after correcting for multiple comparisons) no item from the RRB subdomain differentiated the groups in Module 3. The finding that scores on the RRB subdomain differentiated groups in Module 2 but not Module 3 is of particular interest, as it mimics earlier reported findings that severity scores in the RRB subdomain did not differentiate individuals who were classified as having DS-only from those classified as having DS + ASD. As explained above, there are several possible explanations for this finding, although further research is needed to better understand this phenomenon.

The findings regarding differences in symptom profile and severity between the DS + ASD and DS-only groups have more general implications for the field. In particular, in examining the high cooccurrence of ASD and fragile X syndrome (FXS) [42], we have argued previously that the categorical diagnosis of ASD can hide mechanistically and clinically important differences among individuals with FXS, and between those with FXS+ASD and those with non-syndromic ASD. Moreover, we have provided empirical support for that claim in several studies providing in-depth analysis of both the ADOS-2 and the ADI-R and in multiple samples of different ages and degree of impairments [43,44]. In the present study, too, we have shown that an ASD diagnosis can “mask” different levels of severity and symptom profiles in individuals with DS as a function of the ADOS-2 module administered. This finding, we believe, has less to do with the specific characteristics of the module administered and is, instead, a reflection of that ways in which ASD symptoms are moderated by the phenotype of DS and within-syndrome variability in that phenotype. Simply focusing on whether an individual with DS meets or does not meet criteria for an ASD diagnosis, therefore, could have the consequences of a failure to understand the factors leading to those symptoms or the best approach to treatment to reduce those symptoms.

### 4.4. Limitations

Several limitations should be considered when interpreting results of this study. First, prevalence rates reported are based on a sample of convenience rather than using a population-based sample, although it is important to note that the participants were not recruited based on ASD status. With this in mind, caution should be used when interpreting the prevalence rates reported. Second, participants’ ASD status was determined solely based on the ADOS-2, making it impossible to determine whether the findings reported here will be replicable using other diagnostic measures. Third, based on inclusion criteria for the original study, which required a specific level of language (i.e., a minimum of at least occasional three-word phrase speech), our findings may not be representative of all individuals with DS, such as those who may be minimally verbal or nonverbal. This emphasizes the need for replication of these findings using samples that include individualizing with a range of language abilities, utilizing the full range of ADOS-2 modules. The inclusion of a sample representing the full range of language abilities would allow a better understanding of how ASD symptomatology present differentially among individuals with differing language abilities. Moreover, our findings of higher rates of classification for youth with more limited language abilities, highlights the importance of considering language skills relative to ASD symptomatology; studies focused on these associations in youth with DS who are in the prelinguistic and first words stages of language development are critical to more fully understand the relation between ASD and language skill. Finally, we did not include other non-DS comparison groups. In particular, it would be useful in future studies of ASD in DS to make comparisons with appropriately matched groups of individuals with non-syndromic ASD and ID of a different origin (e.g., fragile X syndrome). Comparisons between individuals with DS + ASD and matched group of individuals with non-syndromic ASD would allow for a deeper understanding of how the DS phenotypes moderates the expression of ASD and the specific phenotypic factors that play a role in that moderation. Although there is a small body of work that examines the differences in presentation of ASD symptomatology among individuals with DS and those with non-syndromic ASD, e.g., [15,18], further research is needed to truly understand which symptoms are attributable to DS phenotype and which are attributable to ASD among individual with DS + ASD. Similarly, there is a fledgling body of work examining differences between individuals with DS + ASD and matched individuals with ID of other etiologies [45]. Given the dearth of research in this area, future research is needed to are needed help identify which aspects of the expression of ASD that are unique to DS rather than common to those with ID.

## 5. Conclusions

The findings reported in the current study further elucidate the prevalence of ASD symptomatology in a sample of individuals with DS, as measured by gold standard diagnostic instrument, the ADOS-2. The findings of this study replicate previously reported findings of increased challenges related to social communication and higher levels of rigid and repetitive behaviors among individuals with DS + ASD in comparison to those with DS-only. We also highlight the contribution of language delays to the classification of ASD in this sample, which underscores previously raised questions regarding the boundary between phenotypic characteristics of DS and true ASD symptomatology. Although this study contributes to the field by examining the prevalence and presentation of ASD in the largest sample of individuals with DS to date, it also demonstrates the need for more research exploring the complexities of diagnosing ASD among individuals with DS.

## Figures and Tables

**Table 1:** Participant demographics for overall sample, Module 2 and Module 3.

	Overall Sample			Module 2			Module 3		
	N	Frequency	%	N	Frequency	%	N	Frequency	%
**Gender (M)**	83	45	54.2	45	28	62.2	38	17	44.7
**Race**	83			45			38		
African American/Black		2	2.4		2	4.4		0	0
Asian/Pacific Islander		1	1.2		1	2.2		0	0
White		58	69.9		31	68.9		27	71.1
Multiple Races		9	10.8		6	13.3		3	7.9
Unknown		12	14.5		5	11.1		7	18.4
Other		1	1.2		0	0		1	2.6
**Ethnicity**	83			45			38		
Hispanic/Latino		16	19.3		8	9.6		8	9.6
**Yearly Income**	81			43			38		
Less than 25,000		5	6.0		3	7.0		2	6.2
25,000–50,000		18	21.7		13	30.2		5	22.2
50,000–75,000		12	14.5		8	18.6		4	14.8
75,000–100,000		13	15.7		5	11.6		8	16.0
100,000–150,000		15	18.1		6	14.0		9	18.5
150,000–250,000		12	14.5		7	16.3		5	14.8
Over 250,000		6	7.2		1	2.3		5	7.4
	**N**	**Mean**	**SD**	**N**	**Mean**	**SD**	**N**	**Mean**	**SD**
**CA (years)**	83	15.60	5.18	45	13.66	5.35	38	17.89	3.93
**Cognitive**									
FSIQ Deviation	71	46.66	11.21	38	40.77	8.53	33	53.44	10.12
NVIQ Deviation	77	50.91	11.29	41	45.85	9.81	36	56.68	10.12
VIQ Deviation	74	41.88	12.60	41	35.30	9.67	33	50.06	10.98
**ADOS-2**									
CSS	83	3.27	2.16	45	3.73	2.19	38	2.71	2.04
SA Severity	83	3.83	2.12	45	4.27	2.05	38	3.32	2.11
RRB Severity	83	2.72	4.27	45	4.31	2.24	38	4.21	2.72
**Adaptive**	65			40			25		
**Functioning**									
Vineland ABC SS		73.69	28.03		75.93	35.00		70.12	9.09

Note: SES = Socioeconomic Status; CA = Chronological Age; FSIQ Deviation = Full Scale IQ Deviation; NVIQ Deviation = Non-Verbal IQ Deviation; VIQ Deviation = Verbal IQ Deviation; CSS = Calibrated Severity Score; SA Severity = Social Affective Severity Score; RRB Severity = Rigid and Repetitive Behavior Severity Score; Vineland ABC SS = Adaptive Behavior Composite Standard Score.

**Table 2:** Means for Participants Characteristics for overall sample, Module 2 and Module 3.

		Overall Sample			Module 2			Module 3		
		N	Mean	SD	N	Mean	SD	N	Mean	SD
CA	DS-only	52	15.14	5.21	24	11.87	4.77	28	17.93	3.78
	DS + ASD	31	16.36	5.14	21	15.69	5.36	10	17.78	4.57
	Total	83	15.60	5.19	45	13.66	5.35	38	17.89	3.93
SB-5 NV Change Sensitive Score	DS-only	47	464.47	13.80	21	455.95	11.63	26	471.35	11.50
	DS + ASD	30	458.07	12.65	20	454.95	12.95	10	464.30	9.87
	Total	77	461.97	13.65	41	455.46	12.15	36	469.39	11.39
Syntactic Complexity	DS-only	51	5.18	2.02	23	3.76	1.61	28	6.35	1.533
	DS + ASD	30	3.38	1.86	20	2.52	1.42	10	5.09	1.40
	Total	81	4.51	2.14	43	3.19	1.63	38	6.02	1.58
Lexical Diversity	DS-only	51	72.71	37.09	23	49.17	32.26	28	92.04	28.98
	DS + ASD	30	50.63	32.38	20	35.50	23.07	10	80.90	26.99
	Total	81	64.53	36.81	43	42.81	28.87	38	89.11	18.54

Note: CA = Chronological age; SB-5 NV Change Sensitive Score = Stanford Binet-5 Non-Verbal Change Sensitive Score.

## Data Availability

The datasets used and/or analyzed for the present paper can be made available upon a reasonable request to the corresponding author.
